# Aldo-Keto Reductases 1B in Endocrinology and Metabolism

**DOI:** 10.3389/fphar.2012.00148

**Published:** 2012-08-02

**Authors:** Emilie Pastel, Jean-Christophe Pointud, Fanny Volat, Antoine Martinez, Anne-Marie Lefrançois-Martinez

**Affiliations:** ^1^CNRS, UMR6293/INSERM U1103, Génétique, Reproduction et Développement, Clermont UniversitéAubière, France

**Keywords:** aldose reductases, adipose tissue, prostaglandins, enterohepatic tissue, metabolism

## Abstract

The aldose reductase (AR; human AKR1B1/mouse Akr1b3) has been the focus of many research because of its role in diabetic complications. The starting point of these alterations is the massive entry of glucose in polyol pathway where it is converted into sorbitol by this enzyme. However, the issue of AR function in non-diabetic condition remains unresolved. AR-like enzymes (AKR1B10, Akr1b7, and Akr1b8) are highly related isoforms often co-expressed with *bona fide* AR, making functional analysis of one or the other isoform a challenging task. AKR1B/Akr1b members share at least 65% protein identity and the general ability to reduce many redundant substrates such as aldehydes provided from lipid peroxidation, steroids and their by-products, and xenobiotics *in vitro*. Based on these properties, AKR1B/Akr1b are generally considered as detoxifying enzymes. Considering that divergences should be more informative than similarities to help understanding their physiological functions, we chose to review specific hallmarks of each human/mouse isoforms by focusing on tissue distribution and specific mechanisms of gene regulation. Indeed, although the AR shows ubiquitous expression, AR-like proteins exhibit tissue-specific patterns of expression. We focused on three organs where certain isoforms are enriched, the adrenal gland, enterohepatic, and adipose tissues and tried to connect recent enzymatic and regulation data with endocrine and metabolic functions of these organs. We presented recent mouse models showing unsuspected physiological functions in the regulation of glucido-lipidic metabolism and adipose tissue homeostasis. Beyond the widely accepted idea that AKR1B/Akr1b are detoxification enzymes, these recent reports provide growing evidences that they are able to modify or generate signal molecules. This conceptually shifts this class of enzymes from unenviable status of scavenger to upper class of messengers.

## Introduction

Aldose reductases (AR) are cytosolic monomeric enzymes, belonging to the aldo-keto reductase (AKR) superfamily. This superfamily encompasses more than 150 NAD(P)(H)-dependent oxidoreductases distributed in all prokaryotic and eukaryotic kingdoms including yeast, plant, invertebrates, and vertebrates. They catalyze the reduction of carbonyl groups from a wide variety of natural or synthetic substrates such as aliphatic and aromatic aldehydes, ketones, keto prostaglandins, ketosteroids, and xenobiotics. Because of overlapping substrates and coenzyme specificities that could lead to confusion between these closely conserved proteins, a nomenclature system for the AKR superfamily has been established based on their structural and genetic properties and is available at www.med.upenn.edu/akr/. Indeed, based on sequence identity, these proteins are divided in 15 families termed AKR1–AKR15, each family having less than 40% amino acid sequence identity with the others. Some families are further subdivided into subfamilies containing proteins with more than 60% sequence identity (Jez et al., 1997; Hyndman et al., 2003). To date, the AKR1 family is the major group encompassing 50 variants of the referring founder protein AKR1A1.

Among the AKR1 family, the AR subgroup (AKR1B) is one of the most characterized because of its involvement in human diseases such as diabetic complications resulting from the ability of AKR1B1 to reduce glucose into sorbitol in a NADPH + H^+^dependent manner. In addition to glucose conversion, AKR1B proteins display multiple other activities including reduction of aldehydes generated by lipid peroxidation, steroids and their derivatives or by-products, retinoids, xenobiotics, and prostaglandins.

The AKR1B subfamily contains several proteins with very high structural similarity to the human former AKR1B1. The AKR1B proteins share more than 65% sequence identity (Table 1). According to their phylogenetic features and their ability to reduce glucose, they can be classified into two subgroups, i.e., AR (AKR1B1–6) and aldose reductase-like proteins (ARLP; Akr1b7–16), respectively.

**Table 1:** **Comparison of protein sequence of AKR1B**.

	% cDNA identity	% Protein identity	% C-ter identity/similarity		Sequences of the 17 C-ter AA residues
**HUMAN**					
AKR1B1^•^	100	100	100	100	A**L**LSCTSH**KDYPFH**E**E**F
AKR1B10*	70	71	35	59	NVLQSSHL**EDYPFD**A**E**Y
AKR1B15	68	68	23	47	DFKEFSHL**EDFPFD**A**E**Y
**MOUSE**					
Akr1b3^•^	80	85	70	76	A**L**MSCAKH**KDYPFH**A**E**V
Akr1b7^ 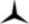 ^	72	71	52	71	D**L**LDARTE**EDYPFH**E**E**Y
Akr1b8*	71	70	23	53	L**L**PETVNM**EEYPYD**A**E**Y
Akr1b16	69	70	35	71	G**L**FAASHN**EDFPFH**A**E**Y
**RAT**					
Akr1b4^•^	82	84	70	76	A**L**MSCAKH**KDYPFH**A**E**V
r-Akr1b10	68	69	35	71	G**L**FAASRN**EDFPFH**S**E**Y
Akr1b13*	76	71	17	53	L**L**PETVNM**EEFPYD**A**E**Y
Akr1b14^ 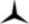 ^	69	68	41	65	G**L**FVTSDE**EDFPFH**E**E**Y

*^•^ or * or ^
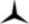
^, corresponding proteins are encoded by ortholog genes; Bold letter, conserved amino acid residues; AA, amino acid*.

To date, three human AKR1B have been characterized: AKR1B1 (human AR; Bohren et al., 1989), AKR1B10 [also designated as human small intestine (HSI) reductase; Cao et al., 1998; Hyndman and Flynn, 1998], and AKR1B15 (Barski et al., 2008; Salabei et al., 2011), which are encoded by genes tandemly arrayed on chromosome 7q33–35. *AKR1B1* seems to be ubiquitously expressed whereas *AKR1B10* expression was only reported in small intestine, colon, liver, and thymus (Cao et al., 1998). A genetic study recently identified a new gene named *AKR1B15* closely related to the *AKR1B1* and *AKR1B10* cluster on chromosome 7, encoding a putative protein sharing 68 and 91% sequence identity with AKR1B1 and AKR1B10, respectively. *AKR1B15* tissue expression has not been explored so far (Barski et al., 2008; Salabei et al., 2011).

Four murine Akr1b have been described: Akr1b3 (murine AR; Gui et al., 1995), Akr1b7 [previously named Mouse vas deferens protein (MVDP); Pailhoux et al., 1990], Akr1b8 [previously named fibroblast growth factor-related protein (FR-1); Donohue et al., 1994], and Akr1b16 (Salabei et al., 2011). Murine AR genes are located on chromosome 6 (locus 6 B1) and their tandem arrangement suggests (as for the three human *AKR1B*) that these four genes arise from an ancestral gene duplication event (Ho et al., 1999; Ruiz et al., 2011). Like *AKR1B1*, *Akr1b3* and *Akr1b16* seem to be ubiquitously expressed (Joshi et al., 2010; Salabei et al., 2011) whereas *Akr1b7* and *Akr1b8* display more restricted tissue distribution: *Akr1b7* is expressed in vas deferens, adrenal glands, gonads, intestine, white adipose tissue, eye, liver, and kidney (Pailhoux et al., 1990; Lau et al., 1995; Tirard et al., 2007; Brunskill et al., 2011; Schmidt et al., 2011). *Akr1b8* expression is detected in testis, heart, adrenal glands, intestine, and liver (Donohue et al., 1994; Lau et al., 1995; Joshi et al., 2010).

Among rat AR, Akr1b4 seems to be ubiquitously expressed whereas the other related proteins have a more restricted expression pattern (MacLeod et al., 2010). Transcripts for *Akr1b14* have been detected in liver, kidney, and adrenals. Those of *r-Akr1b10* share this tissue expression pattern but are also found in brain, heart, and lungs (Endo et al., 2010b). *Akr1b13* expression has been observed at mRNA level in almost all organs, except brain and liver but the protein remains undetectable in small intestine and colon (Endo et al., 2011).

Several studies allowed identification of *Akr1b4*, *Akr1b13*, and *Akr1b14* as the orthologs of mouse *Akr1b3*, *Akr1b8*, and *Akr1b7*, respectively (Barski et al., 2008; Endo et al., 2011). This phylogenetic analysis between rat and mouse AR has some limits: Akr1b14 and Akr1b7 do not exactly display the same expression pattern. Indeed, vas deferens is the major site of Akr1b7 expression whereas Akr1b14 is barely detectable in this tissue. Despite high sequence identity, the two promoters differ by a short 77-bp region absent in rat sequence which confers vas deferens targeted expression and androgen responsiveness to the *Akr1b7* gene (Val et al., 2002).

Identification of the mechanisms regulating expression of AKR1B/Akr1b is a necessary step to understand their physiological functions. Nevertheless, the precise identification and determination of spatial distribution of AKR1B/Akr1b in healthy tissues remains a difficult task, as it requires powerful and specific immunological or nucleic probes to allow discrimination between these closely related isoforms. For that reason, we designed immunological probes targeting the most divergent domain among these enzymes that encompass the 17 C-terminal amino acid residues (Table 1). Indeed, using this C-terminal region critical to substrate specificity (Bohren et al., 1992), we developed immunological tools discriminating Akr1b7, Akr1b3, and Akr1b8 isoforms (Lefrançois-Martinez et al., 2004). Similarly, the most discriminating nucleotide probes or primers should be carefully designed from 5′- to 3′-untranslated regions while open reading frame should be avoided whenever possible. Knowing that in mammals, AR genes (i.e., human *AKR1B1* gene, murine *Akr1b3*, and rat *Akr1b4*) display a broad tissue expression pattern, it can be assumed that in normal conditions, most of their activities are involved in maintaining general cellular homeostasis through osmotic regulation or detoxification processes.

This review will focus on the most characterized AKR1B proteins: AKR1B1, Akr1b3, Akr1b7, Akr1b8, and AKR1B10. To date many *in vitro* and *ex vivo* studies have examined and carefully described their enzymatic activities and defined their substrate specificities. These studies have enlightened some redundancy in substrate specificities that can be confusing when considering that various isoforms may coexist in the same tissue. In light of these enzymatic data, one of the most challenging issue regarding AKR1B enzymes would now be to explore their distinct biological functions in specific physiological or pathological processes. The focus of this review is to integrate most recent data on specific regulations of AR genes with enzymatic and functional data, in selected organs involved in endocrine and metabolic function, i.e., the adrenal gland, enterohepatic tissue, and white adipose tissue.

## AKR1B and Adrenal Endocrine Function

The adrenal gland has two anatomically and functionally different components: the outer cortex which provides mineralocorticoids from the *zona glomerulosa*, glucocorticoids from the *zona fasciculate*, and the inner medulla in which chromaffin cells produce adrenal catecholamines, i.e., epinephrine and norepinephrine and various neuropeptides.

Acute and chronic adrenal cortex steroidogenesis is regulated mainly through activation of the cAMP-dependent protein kinase (PKA) signaling pathway mediated by the pituitary adrenocorticotropin hormone (ACTH).

Excess levels of glucocorticoid in the plasma in turn induce a negative feedback on ACTH production resulting in blunted ACTH-dependent steroidogenesis in the adrenal gland. This blockade of the hypothalamic-pituitary-adrenal axis can be experimentally recapitulated by dexamethasone treatment (a synthetic glucocorticoid). In adrenals, cAMP-induced PKA activation results at least in the phosphorylation of transcription factors such as steroidogenic factor 1 (SF-1), CCAAT Enhancer Binding Protein (C/EBP), and cAMP response element-binding protein. These, in turn stimulate transcription of genes encoding steroidogenic enzymes and proteins responsible for cholesterol metabolism, mobilization and transport. Therefore, adrenal steroidogenesis is strongly associated with production of endogenous harmful lipid aldehyde by-products including isocaproaldehyde (4-methylpentanal) derived from cholesterol side chain cleavage (the first step of steroid synthesis) and 4-hydroxynonenal (4-HNE). Interestingly, previous studies have established that the adrenal gland is one of the major sites for human and murine AR expression (Lau et al., 1995; Hyndman and Flynn, 1998).

### Akr1b8/AKR1B10: Expression profile, detoxification function (tables 2 and 3)

*Akr1b8* messenger was observed by *in situ* hybridization analysis in fetal and adult murine adrenal cortex and remained undetected in the medulla (Lau et al., 1995). Nevertheless, dexamethasone-induced ACTH suppression had no effect on Akr1b8 protein levels (Lambert-Langlais et al., 2009) suggesting that its biological function would not be associated to the ACTH-dependent steroidogenic activity. Indeed, isocaproaldehyde reductase activity was abolished in adrenocortical Y1 cells lacking Akr1b7 (due to stable antisense expression) in the presence of unaltered Akr1b8 protein levels (Lefrancois-Martinez et al., 1999). Accordingly, isocaproaldehyde accumulated and resulted in cellular toxicity despite the presence of Akr1b8. On the basis of these functional studies, of its enzymatic constants and constitutive expression, Akr1b8 does not appear to be the major isocaproaldehyde reductase in the adrenal cortex (Martinez et al., 2001). Although all murine AR exhibit 4-HNE reductase activity, Akr1b8 seems to be the most efficient HNE reductase in mouse tissues (Srivastava et al., 1998; Martinez et al., 2001). This suggests that adrenocortical expression of Akr1b8 could be dedicated to detoxification of aldehyde lipids that are present in large amounts in the cortex (Burczynski et al., 1999). Nevertheless, functional demonstration remains to be established.

*AKR1B10* mRNA has been detected in adrenal glands using a human RNA Master Blot, but to date, there is no information available on its *in situ* localization and transcriptional control in this organ (Hyndman and Flynn, 1998). Although both AKR1B1 and AKR1B10 human AR display 4-HNE reductase activity, AKR1B10 exhibits a higher activity and product turn over than AKR1B1 (Shen et al., 2011). Both AKR1B1 and AKR1B10 isoforms are expressed in the human adrenal gland and *ex vivo* studies revealed that they also share the ability to reduce isocaproaldehyde (Hyndman and Flynn, 1998). Nevertheless, in a comparative enzymatic study, Hara and colleagues showed that AKR1B1 had a more effective isocaproaldehyde reductase activity than AKR1B10, suggesting that the latter was unlikely to play a major role in the detoxification of steroidogenic by-products (Endo et al., 2009b).

Since its identification in human hepatocellular carcinoma, AKR1B10 was shown to be differentially expressed in other tumor and normal tissues (Cao et al., 1998; Fukumoto et al., 2005; Yoshitake et al., 2007; Rajkumar et al., 2010). AKR1B10 was suggested to be a biomarker of smoker’s non-small cell lung carcinomas (Fukumoto et al., 2005) and to be involved in drug resistance (Matsunaga et al., 2012).

Human adrenocortical carcinoma (ACC) are rare malignant tumors generally associated with a poor prognosis (Libè et al., 2007). In order to identify molecular predictors of malignancy and of survival, 153 unilateral adrenocortical tumors were studied by microarray (http://www.ebi.ac.uk/arrayexpress, experiment E-TABM-311). Unsupervised clustering analysis was performed which allowed robust discrimination of malignant and benign tumors. On the basis of this analysis, AKR1B10 was not found to be associated with the ACC group (de Reyniès et al., 2009).

### Akr1b7: Expression profile, detoxification function, and paracrine action (tables 2 and 3)

High levels of *Akr1b7* transcripts were initially observed by *in situ* hybridization in fetal and adult murine adrenal cortex but were undetectable in the medulla (Lau et al., 1995). We confirmed these results by immunohistochemistry experiments which allowed us to further restrict Akr1b7 expression to the *zona fasciculata* (Aigueperse et al., 1999). *In vivo*, ACTH suppression with dexamethasone treatment resulted in a marked decrease of *Akr1b7* mRNA levels that were restored when the treated mice were injected with exogenous ACTH. This ACTH or cAMP-induced Akr1b7 transcription was blocked by a PKA inhibitor (H89) in the murine adrenocortical ATC and Y1 cell lines, respectively (Aigueperse et al., 1999; Ragazzon et al., 2006).

**Figure 1:**
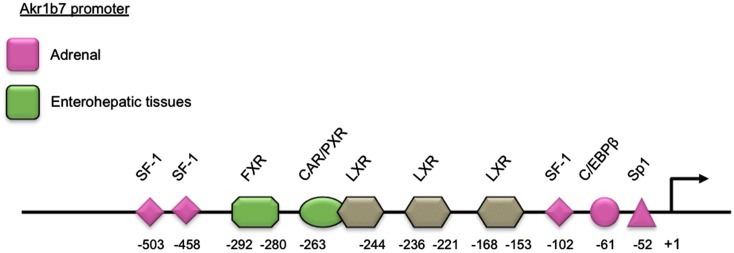
**Schematic representation of the *Akr1b7* promoter**. The DNA binding site for transcription factors and nuclear receptors required for the *Akr1b7* specific expression in adrenal gland (pink boxes) and in enterohepatic tissue (green boxes) are shown. LXR binding sites are involved in both adrenal and intestine *Akr1b7* expression.

Adrenal expression and ACTH regulation of Akr1b7 are supported by three SF-1 binding sites and other *cis*-elements located in the 5′-flanking regulatory region of the gene. Using transgenic mice and cell transfection experiments, we delimited a cryptic SF-1 response element (SFRE) 102 bp upstream of the transcription start site. This SFRE supported basal adrenal promoter activity. Two other SFREs were identified further upstream. The site at −458 was a *bona fide* SFRE playing an essential role for both basal promoter activity and cAMP responsiveness whereas the site at −503 conferred intrinsic cAMP-sensing ability (Martinez et al., 2003; Val et al., 2004). Two other binding sites for the *trans*-acting factors Sp1 and C/EBPβ at position −52 and −61, respectively, also contributed to the transcriptional cAMP responsiveness (Aigueperse et al., 2001; Table 2 and Figure 1).

**Table 2:** **Localization and regulation of AKR1B expression in adrenal gland**.

Isoforms	Localization	Analyses	Control by ACTH/cAMP	Transcriptional regulators	Reference
**HUMAN**					
AKR1B1	Cortex	IHC, RNA Master blot	+	n.d.	^a,b^
AKR1B10	Adrenal^ 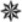 ^	RNA master blot	n.d.	n.d.	^b^
AKR1B15	n.d.	n.d.	n.d.	n.d.	–
**MOUSE**					
Akr1b3	Cortex + medulla	WB	No	No	^a,c^
Akr1b7	Cortex	NB, WB, IHC, ISH	+	Sp1, C/EBPβ, SF-1	^a,c,d,e,f,g^
Akr1b8	Cortex	WB, ISH	No	No	^a,c,d^
Akr1b16	n.d.	n.d.	n.d.	n.d.	–
**RAT**					
Akr1b4	Cortex	RT-PCR, IHC, WB	n.d.	n.d.	^h,i^
Akr1b13	Adrenal^ 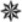 ^	RT-PCR	n.d.	n.d.	^h^
Akr1b14	Cortex	ISH, WB	+	n.d.	^j^

*^
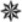
^, Intra-adrenal tissular localization was not specified; n.d., not determined; NB, Northern blot; WB, Western blot; RT-PCR, reverse transcription-polymerase chain reaction; ISH, *in situ* hybridization; IHC, immunohistochemistry*.

*^a^Lambert-Langlais et al. (2009), ^b^Hyndman and Flynn (1998), ^c^Martinez et al. (2001), ^d^Lau et al. (1995), ^e^Aigueperse et al. (1999), ^f^Val et al. (2004), ^g^Aigueperse et al. (2001), ^h^Endo et al. (2009a), ^i^MacLeod et al. (2010)*.

Aldose reductases are able to reduce isocaproaldehyde that is produced in large amount in the adrenal cortex during steroidogenesis. Indeed, the first step of steroidogenesis is the removal of the cholesterol side chain by the P450scc enzyme, resulting in the formation of pregnenolone and isocaproaldehyde. Furthermore, isocaproaldehyde is a cytotoxic aldehyde whose accumulation in Y1 cells decreased their viability (Lefrancois-Martinez et al., 1999). *In vitro* studies revealed that Akr1b3, Akr1b7, and Akr1b8 all had the ability to reduce isocaproaldehyde. Their kinetic constants suggested that isocaproaldehyde was a major substrate for Akr1b3 and Akr1b7, and a poor substrate for Akr1b8 (Martinez et al., 2001; Table 3). Akr1b7 silencing in Y1 adrenocortical cells disrupted cAMP-induced isocaproaldehyde reductase activity. This strongly suggested that Akr1b7, rather than Akr1b3, was the main isocaproaldehyde reductase in the adrenal gland (Lefrancois-Martinez et al., 1999). These observations further indicate that ACTH not only coordinates expression of enzymes responsible for the biosynthesis of steroids, but also of non-steroidogenic enzymes involved in the detoxification of reactive aldehydes generated during steroidogenesis.

**Table 3:** **Kinetic constants of AKR1B toward 4-hydroxynonenal, isocaproaldehyde, and prostaglandin H2**.

Substrates	4-Hydroxynonenal		Isocaproaldehyde		Prostaglandin H2^e^	
	*K*_m_ (μM)	*K*_cat_ (s^−1^)	*K*_m_ (μM)	*K*_cat_ (s^−1^)	*K*_m_ (μM)	*V*_max_ (nmol/min/mg)
**HUMAN**						
AKR1B1	716^a^	0.84^a^	1^b^	0.66^b^	1.9	44
AKR1B10	31^a^	2.01^a^	330^c^	0.72^c^	No activity	
AKR1B15	n.d.	n.d.	n.d.	n.d.	n.d.	
**MOUSE**						
Akr1b3	665^a^	0.82^a^	62^d^	1.3^d^	9.3	26
Akr1b7	256^a^	0.1^a^	320^d^	0.38^d^	3.8	53.4
Akr1b8	230^a^	3.18^a^	71^d^	0.03^d^	No activity	
Akr1b16	n.d.	n.d.	n.d.	n.d.	n.d.	
**RAT**						
Akr1b4	33^f^	0.23^f^	n.d.	n.d.	n.d.	
r-Akr1b10	1.6^g^	0.05^g^	11^g^	0.03^g^	n.d.	
Akr1b13	30^f^	0.18^f^	n.d.	n.d.	n.d.	
Akr1b14	7.6^h^	0.02^h^	16^h^	0.03^h^	n.d.	

*n.d., not determined*.

*References (for comparison data were converted in μM for the *K*_m_ and in s^−1^ for the *K*_cat_): ^a^Joshi et al. (2010), ^b^Matsuura et al. (1996), ^c^Martin and Maser (2009), ^d^Martinez et al. (2001), ^e^Kabututu et al. (2009), ^f^Endo et al. (2009a), ^g^Endo et al. (2010b), ^h^Endo et al. (2010a)*.

In a previous study, Madore and colleagues proposed that AKR1B5, initially characterized as the bovine 20α-hydroxysteroid dehydrogenase, ensured prostaglandin F2α synthase (PGFS) activity in the endometrium (Madore et al., 2003). Thereafter, we have established by *in vitro* studies, that this property could be extended to other but not all AKR1B enzymes. Indeed, AKR1B1, Akr1b3, and Akr1b7 were shown to catalyze the reduction of prostaglandin H_2_ (PGH_2_) into PGF_2α_ (Table 3). In contrast, Akr1b8 and AKR1B10 recombinant proteins were devoid of this PGH_2_ 9-,11-endoperoxide reductase activity. This activity has not been investigated for Akr1b16, AKR1B15, and rat AR so far. Importantly, based on their kinetic parameters, recombinant AKR1B1, Akr1b3, and Akr1b7 displayed better PGF synthase activities than the previously characterized PGF synthases in mammals (Kabututu et al., 2009).

Prostaglandins are paracrine/autocrine cell mediators sharing a common precursor, PGH_2_, which is synthesized from free arachidonic acid by the cyclooxygenases type 1 (COX-1) or type 2 (COX-2). COX-1 is regarded as a constitutively expressed enzyme. COX-2, on the other hand, is undetectable in most tissues in basal conditions but can be induced by various mitogenic agents and inflammatory stimuli (Ramsay et al., 2003). Following these observations, we carefully examined the PGF_2α_ biosynthetic pathway in the adrenal gland (Lambert-Langlais et al., 2009).

PGF_2α_ was secreted by both cortical (steroidogenic cells) and medullary (chromaffin cells) compartments of the adrenal gland. In primary adrenocortical cell culture, PGF_2α_ release was induced 2.5-fold by ACTH exposure which was correlated with ACTH-responsiveness of both COX-2 and Akr1b7. Using over- and down-expression strategies in cell lines, we demonstrated the pivotal role of Akr1b7 in ACTH-induced PGF_2α_ release and its functional coupling with COX-2. In the adrenal medulla, PGF_2α_ production seemed to be resulting from the coordinated activities of Akr1b3 and COX-1. In the adrenal, expression of the PGF_2α_ specific receptor (FP) was restricted to chromaffin cells, suggesting that both autocrine (within the medulla) and paracrine (between steroidogenic and chromaffin cells) mechanisms were relaying PGF_2α_ action. Indeed in the chromaffin cell line MPC862L, PGF_2α_ was able to repress both basal and glucocorticoid-induced dopamine release. By comparing PGF_2α_-responsiveness of isolated cells and whole adrenal cultures, we demonstrated that PGF_2α_ repressed glucocorticoid secretion by an indirect mechanism involving a decrease in catecholamine release, which in turn decreased adrenal steroidogenesis.

**Figure 2:**
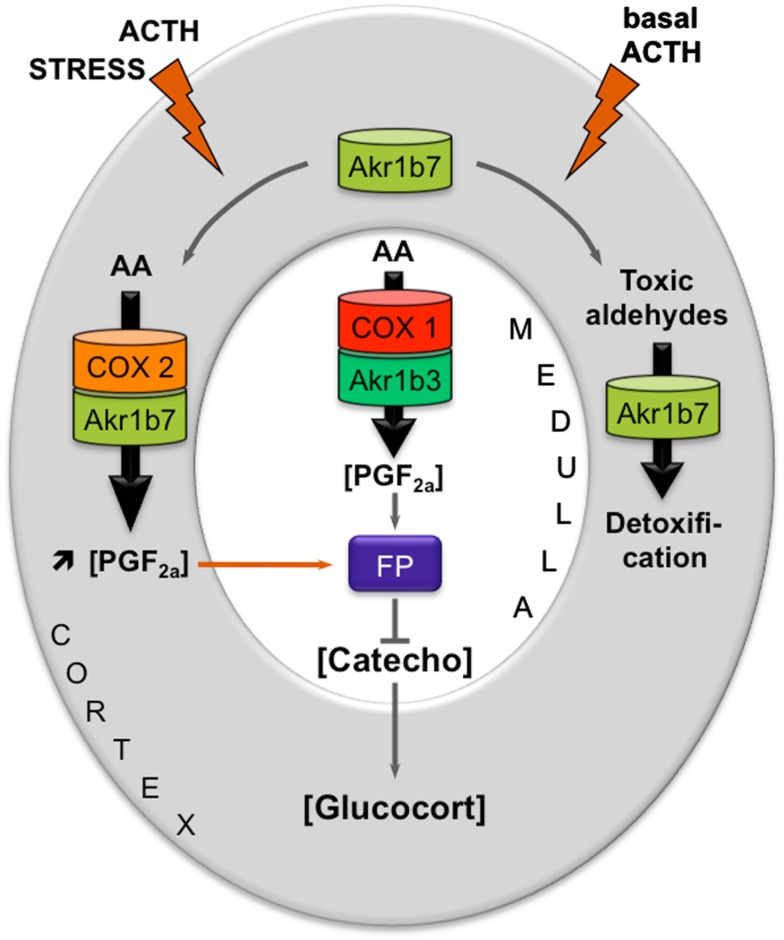
**Proposed model integrating dual functions of aldose reductases in the regulation of adrenal endocrine functions**. Free arachidonic acid (AA) is metabolized into PGH_2_ by COX enzymes and then converted into PGF_2α_ by PGFS of the AKR1B family. FP receptor expression is restricted to the medullary zone. PGF_2α_ synthesized in both the cortex and medulla thus signals in an autocrine/paracrine manner on chromaffin cells. This inhibits catecholamines production. Catecholamines produced in the medulla normally stimulate glucocorticoids release by the cortex. Decreased catecholamines production in response to PGF_2α_ stimulation thus results in a decrease in glucocorticoids production. The differential expression and regulation of both COX and AKR1B enzymes within the adrenal zones could allow the adjustment of PGF_2α_ production to limit stress response or control basal steroidogenesis by finely tuning glucocorticoids secretion. In basal conditions, chromaffin cells of the medullary zone constitutively secrete PGF_2α_, through the functional coupling between COX-1 and possibly the PGFS Akr1b3. Under stress conditions, the resulting ACTH surge induces COX-2 expression and sustains Akr1b7 levels in the cortex. The functional coupling between COX-2 and Akr1b7 triggers a PGF_2α_ surge that could act as a local paracrine feedback to limit catecholamine-mediated glucocorticoid release. After the stress response has ended, COX-2 returns to undetectable levels. The coupling between Akr1b7 and COX-2 does not take place. Akr1b7 then functions only as a detoxifying enzyme of the harmful aldehydes produced under chronic/basal stimulation of steroidogenesis. Catecho, catecholamines; Gluco, glucocorticoids.

These results allowed us to propose a new mechanism for an intra-adrenal feedback loop, in which AR play a pivotal role in the regulation of adrenal endocrine functions (Figure 2). The mechanism that we proposed was the following: (1) In basal conditions, PGF_2α_ is constitutively secreted by chromaffin cells (by the coupling of COX-1 and Akr1b3), thus regulating catecholamines production and also limiting their paracrine action on steroidogenesis. (2) During a stress situation, ACTH transiently induces COX-2 and Akr1b7 expression, which results in PGF_2α_ production inside the cortex. PGF_2α_ produced in the cortex then represses catecholamines release by the medulla, via a paracrine action on its FP receptor. Decreased catecholamines release in turn reduces the effect of ACTH on glucocorticoids production (Lambert-Langlais et al., 2009).

### Akr1b3/AKR1B1 expression profile, detoxification function, and paracrine action

By using different adrenal cell lines, we managed to detect Ak1b3 protein in the adrenocortical Y1 cells, and in the chromaffin MPC862L cells. These results confirmed that, unlike other murine AR, Akr1b3 is constitutively expressed in the whole adrenal gland (Lambert-Langlais et al., 2009). In Y1 adrenocortical cell line and during *in vivo* hormonal manipulations, cAMP appeared to have no significant effect on Akr1b3 expression suggesting that its expression was insensitive to ACTH (Martinez et al., 2001; Table 2).

Akr1b3 is also involved in the detoxification of toxic carbonyls. Even if Akr1b7 is the main isocaproaldehyde reductase and Akr1b8 the principal 4-HNE reductase, Akr1b3 is also able to reduce these toxic compounds. This suggests that Akr1b3 can take part in the elimination of these compounds in basal physiological conditions (Lefrancois-Martinez et al., 1999; Martinez et al., 2001). Moreover, Akr1b3 is constitutively expressed in cortical and medullary cells, where it could be coupled to COX-1 to synthesize PGF_2α_ (see above paragraph).

Despite its expected participation in the elimination of toxic compounds and in the production of signal molecules (PGF_2α_), there is no report of adrenal dysfunction in mice lacking Akr1b3 (*Akr1b3*^−/−^; Aida et al., 2000; Ho et al., 2000). This absence of adrenal phenotype may result from functional redundancy between the different family members present in the gland (Akr1b7 and Akr1b8).

*AKR1B1* expression has initially been detected using a RNA master blot in human adrenal gland (Hyndman and Flynn, 1998). More recently, immunohistochemistry experiments allowed us to assign AKR1B1 to the cortical compartment of the gland (Lambert-Langlais et al., 2009). In NC1-H295, a human adrenocortical tumor cell line, *AKR1B1* mRNA levels were induced by forskolin (adenylyl cyclase inducer) treatment (Lefrançois-Martinez et al., 2004). This suggested that similarly to *Akr1b7*, *AKR1B1* gene expression could be sensitive to ACTH control. However, the molecular mechanisms and *cis*-acting elements ensuring ACTH/cAMP responsiveness of *AKR1B1* gene have remained unexplored.

For years, AKR1B1 had been considered as the major isocaproaldehyde reductase in the adrenal gland (Matsuura et al., 1996). The NADPH-dependent isocaproaldehyde reductase activity harbored by AKR1B1 was inhibited by tolrestat while the murine isocaproaldehyde reductase Akr1b7 was insensitive to this pharmacological inhibitor (Matsuura et al., 1996; Lefrancois-Martinez et al., 1999). We demonstrated that AKR1B1 was also endowed with 9-,11-endoperoxide reductase activity (Kabututu et al., 2009). The conversion of PGH_2_ into PGF_2α_, catalyzed by AKR1B1, strictly dependent on the presence of NADPH, was inhibited by tolrestat whereas Akr1b7 PGFS activity was insensitive to AR inhibitors.

We showed that in the normal human adrenal gland, AKR1B1 and COX-2 were co-localized in steroidogenic cortical cells (Lambert-Langlais et al., 2009). Therefore, the human adrenal cortex could also have the potential to produce PGF_2α_ in response to ACTH. With respect to their hormonal regulation in the adrenal cortex and their reductase activity toward common substrates, we have postulated that AKR1B1 could be considered as a functional ortholog of Akr1b7 in the human adrenal cortex (Lefrançois-Martinez et al., 2004). The possibility that the PGFS activity of AKR1B1 could be involved in an intra-adrenal feedback loop between endocrine activities of cortical and medullary compartments in human adrenal gland remains to be explored.

Given the high expression of AKR1B1 in the adrenal cortex we evaluated alterations in its expression in association with human adrenal disorders. The relative abundance of *AKR1B1* mRNA was decreased in ACCs when compared to benign tumors, Cushing’s hyperplasia, or normal adrenals (Lefrançois-Martinez et al., 2004). These data provided evidence that expression of AKR1B1 was decreased in adrenocortical cancer. This was further confirmed by the unsupervised clustering analysis of the human adrenal tumors transcriptome performed by de Reyniès et al. (2009), indicating that decreased expression of AKR1B1 correlated with malignancy for the molecular diagnosis of adrenal tumors.

## AKR1B and Glucido-Lipidic Homeostasis

### AKR1B in enterohepatic tissues

Excessive nutrient intake is detrimental for cells and tissues. In mammals, the liver converts excess dietary carbohydrates into triglycerides through *de novo* lipogenesis. Two transcription factors, carbohydrate-responsive element-binding protein (ChREBP) and sterol responsive element-binding protein-1c (SREBP-1c) emerged as major mediators of glucose and insulin action in the control of both glycolysis and lipogenesis in the liver. The liver X receptors (LXR) are oxysterol activated transcription factors acting as important metabolic regulators of the lipogenic pathway. Indeed, LXRs ensure the transcriptional control of SREBP-1c in response to insulin and of ChREBP. Moreover, direct targets of LXRs include other lipogenic genes such as *fatty acid synthase* (*Fas*) and *stearoyl-CoA desaturase 1* (*SCD1*; Chen et al., 2004; Postic and Girard, 2008; Kim et al., 2009). The farnesoid X receptor (FXR)/bile acid receptor is another nuclear receptor that plays an important role in maintaining bile acid, lipid, and glucose homeostasis since its activation has been shown to lower blood triglyceride and cholesterol levels and to improve insulin sensitivity in diabetic mouse models (Zhang and Edwards, 2008). In enterocytes, FXR was shown to protect against the cytotoxic effects of bile acids by increasing expression of binding proteins and transporters (Schmidt and Mangelsdorf, 2008).

#### Akr1b8/AKR1B10: From cell detoxification to lipid synthesis

Previous analyses performed by RNAse protection assays reported very weak levels of *Akr1b8* transcript (Lau et al., 1995) in the mouse intestine and absence of expression in the adult liver. These observations were partially revised by Joshi et al. (2010) who showed high *Akr1b8* expression all along the adult intestinal tract and moderate expression in the liver. *Akr1b8* expression was initially described to be up-regulated during the early phase of the cell cycle and induced by growth promoting agents [fibroblast growth factor (FGF) and epidermal growth factor (EGF)], as well as by hypertonic stress (Donohue et al., 1994; Hsu et al., 1997). Although *Akr1b8* displayed a strong expression in mouse colon cancer cells (Joshi et al., 2010) and was suggested to be a target gene for NF-E2 related factor 2 (Nrf2) transcription factor that controls intestinal detoxification response (Varady et al., 2011), its expression and regulation in healthy liver and intestine remained elusive. A recent study investigating bile acids impacts on *Akr1b7* expression showed that enterohepatic expression of *Akr1b8* was insensitive to bile acids (Schmidt et al., 2011). Akr1b8 enzymatic activities were extensively studied regarding its ability to reduce the highly reactive 4-HNE produced during peroxidation of polyunsaturated fatty acids (Srivastava et al., 1998). *In vitro* enzymatic studies reported that multiple members of the AKR1B group, including Akr1b8, were endowed with the ability to reduce phospholipid aldehydes derived from lipid peroxidation. Compared with AKR1B1 or Akr1b7, Akr1b8 activity was more efficient with the reduction of short chain aldehydes such as 1-palmitoyl-2-arachidonoyl-*sn*-glycero-3-phosphocholine and 1-pamitoyl-2-arachidonoyl-*sn*-glycero-3-phosphoglycerol (Spite et al., 2007). In line with the high lipid metabolic activities in Akr1b8 producing sites, it was proposed that Akr1b8 was acting physiologically as a scavenger of toxic aldehydes derived from peroxidation of endogenous or dietary lipids.

Beside its aldehyde reductase activity, Akr1b8 was shown to associate with the lipogenic acetyl-CoA carboxylase α (ACCA) in murine colon cancer cells (Joshi et al., 2010). ACCA is a rate-limiting enzyme of *de novo* synthesis of fatty acids, catalyzing the formation of malonyl-coA by ATP-dependent carboxylation of acetyl-coA. This interaction protects ACCA from proteasomal degradation and consequently allows increased fatty acid synthesis that leads to production of lipid second messengers that promote cell proliferation (Chajès et al., 2006). Whether Akr1b8 is also involved in the stability of ACCA in healthy enterocytes has not yet been determined. In order to associate gene signature with metabolic network, a genetic study integrating quantitative trait locus (QTL) mapping and network modeling led to knock-out (KO) the three corresponding identified genes, among which *Akr1b8* (Derry et al., 2010). *Akr1b8*^−/−^ mice phenotype was very briefly described in this study suggesting a more specific perturbation in fat tissue homeostasis rather than in the liver or intestine (see below).

**Figure 3:**
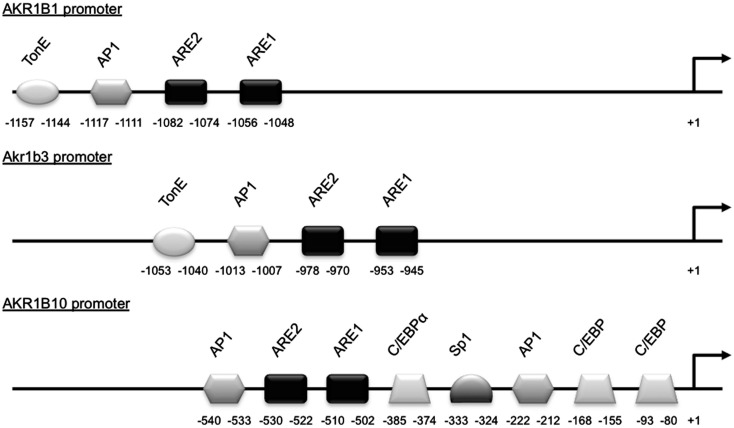
**Schematic representation of the *AKR1B1*, *Akr1b3*, and *AKR1B10* promoters**. *Cis*- and *trans*-acting factors shown to be involved in stress responsiveness are indicated. TonE, tonicity response element; AP1, activator protein-1 binding site; ARE, antioxidant response element; C/EBP, CCAAT enhancer binding protein binding site; Sp1, selective promoter factor 1 binding site.

The *AKR1B10* gene has recently been determined as the human ortholog of *Akr1b8* (Joshi et al., 2010). Its expression was primarily detected in healthy colon, small intestine, and liver (Cao et al., 1998) and was found up-regulated with their corresponding tumorigenic transformation (Cao et al., 1998; Yan et al., 2007; Heringlake et al., 2010; Liu et al., 2012). This was also observed in lung and breast cancers suggesting that AKR1B10 overexpression could be associated with a broader tumor phenotype. In human hepatocarcinoma, insulin or EGF enhanced *AKR1B10* expression through the activator protein-1 (AP1) mitogenic signaling. This enhanced tumor development and progression through elimination of cytotoxic carbonyls and promotion of lipogenesis (Liu et al., 2012). The treatment of human colon cancer SW-480 and HT-29 cell lines with proteasome inhibitors known to increase the expression of Nrf2-regulated genes induced AKR1B10 expression suggesting that AKR1B10 could be a target of the Nrf2 transcription factor (Ebert et al., 2011). Nrf2 responsiveness of *AKR1B10* gene was further demonstrated by co-transfection experiments of *AKR1B10* promoter luciferase reporter constructs in human lung adenocarcinoma cell lines (Nishinaka et al., 2011; Figure 3).

AKR1B10 was the first AKR1B protein which was demonstrated to enhance cell proliferation and promote cell survival through its ability to interact with and stabilize ACCA in breast cancer cells (Ma et al., 2008) and in colon cancer cells (Wang et al., 2009). AKR1B10 mediates ACCA stability through physical association. This was shown to affect fatty acid/lipid synthesis, mitochondrial function, and oxidative status. Identification of Akr1b8/AKR1B10 protein domain interacting with ACCA would be helpful to predict this characteristic for other AKR1B proteins and to develop targeted therapeutic strategies for the modulation of *de novo* fatty acid synthesis in cancer cells.

We are exposed daily to α,β-unsaturated aldehyde and *trans*-2-hexenal through food and drink consumption (Stout et al., 2008). This persistent exposure to electrophilic carbonyls requires an effective defense system to protect intestinal cells from irreversible damage. Similarly to Akr1b8, *in vitro* enzymatic studies on AKR1B10 demonstrated a highly specific ability to reduce physiological levels of 4-HNE and other alpha and beta-unsaturated carbonyls in both their free or glutathione-conjugated form (Martin and Maser, 2009; Zhong et al., 2009). Expression of AKR1B10 may reflect the requirement for intestinal cells to protect against the dietary, lumen microbial and lipid-derived carbonyls.

**Figure 4:**
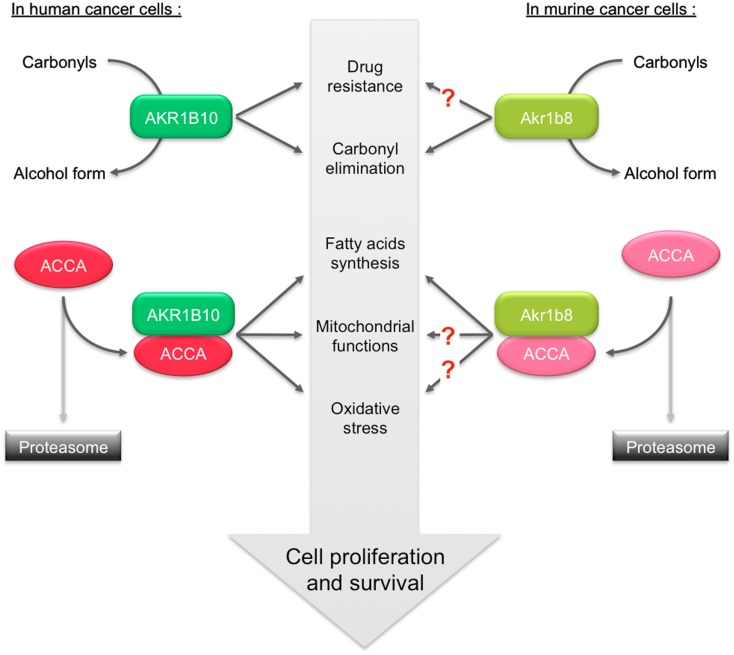
**Role of AKR1B10 and Akr1b8 in the regulation of lipid synthesis and contribution in promoting cancer**. In human cancer cells, ACCA is protected against ubiquitination-proteasome dependent degradation through its association with AKR1B10. Stabilization of ACCA increases fatty acids synthesis. This protects cell from apoptosis by preventing depletion of phospholipids, mitochondrial membrane lesions, and oxidative stress. The dual capacity of AKR1B10 to stimulate lipogenesis and to detoxify carbonyls and drugs thus contributes to enhance cell proliferation/survival. In murine cancer cells, Akr1b8 was also shown to stabilize ACCA thereby modulating fatty acid synthesis. The impact of Akr1b8-mediated ACCA stabilization on mitochondrial function and oxidative stress is still unknown.

Although physiological integration of all these data remains difficult, this suggests that the beneficial role of Akr1b8/AKR1B10 in the protection of healthy cells against lipid peroxidation could switch, in the case of cancer, into a deleterious role promoting cell proliferation through stabilization of ACCA (Figure 4).

#### Akr1b7: Improving (liver) metabolic capacity

##### A target for metabolic nuclear receptors (figure 1 and table 4)

**Table 4:** **Expression of aldose reductases in liver and small intestine**.

Isoforms	Liver	Small intestine	Analyses	Tissue-specific transcriptional regulators	Reference
**HUMAN**					
AKR1B1	+	+	NB, WB	Nrf2	^a,b,c^
AKR1B10	+	+	NB, WB, IHC	AP1, Nrf2	^a,c,d,e,f,g,h^
AKR1B15	n.d.	n.d.	n.d.	n.d.	–
**MOUSE**					
Akr1b3	+	+	RT-PCR	Nrf2	^i,k^
Akr1b7	+	+	RNase protection, RT-qPCR, WB	LXR, CAR, PXR, FXR	^j,l,m,n,o,p,q^
Akr1b8	+	+	RT-PCR, NB	Nrf2	^i,r,s,t^
Akr1b16	+	n.d.	RT-PCR	n.d.	^s^
**RAT**					
Akr1b4	+	+	RT-PCR, WB	n.d.	^u,v^
r-Akr1b10	+	−	RT-PCR	n.d.	^w^
Akr1b13	+	+	RT-PCR, WB	n.d.	^u,w^
Akr1b14	+	−	RT-PCR	n.d.	^u,w^

*n.d., not determined; NB, Northern blot; WB, Western blot; RT-PCR, reverse transcription-polymerase chain reaction*.

*^a^Cao et al. (1998), ^b^O’Connor et al. (1999), ^c^Ebert et al. (2011), ^d^Fukumoto et al. (2005), ^e^Martin et al. (2006), ^f^Heringlake et al. (2010), ^g^Liu et al. (2012), ^h^Nishinaka et al. (2011), ^i^Joshi et al. (2010), ^j^Lau et al. (1995), ^k^Nishinaka and Yabe-Nishimura (2005), ^l^Volle et al. (2004), ^m^Schmidt et al. (2011), ^n^Ge et al. (2011), ^o^Aigueperse et al. (1999), ^p^Lambert-Langlais et al. (2009), ^q^Martinez et al. (2001), ^r^Donohue et al. (1994), ^s^Salabei et al. (2011), ^t^Rangasamy et al. (2004), ^u^Endo et al. (2009b), ^v^MacLeod et al. (2010), ^w^Endo et al. (2010b)*.

Several studies reported specific *Akr1b7* expression in the small intestine (Volle et al., 2004; Ge et al., 2011; Schmidt et al., 2011) within the epithelial cells of the villi (Lau et al., 1995) with a decreasing gradient from the duodenum to the ileum. This expression has been shown to be under the control of oxysterol activated LXR/retinoic X receptor (RXR) heterodimers acting through three LXR response elements (LXRE) located in the *Akr1b7* proximal promoter (LXRE3: −259 to −244, LXRE1: −236 to −221, LXRE2: −153 to −168 from the transcription start site). Two of these *cis*-acting elements were specific for regulation by the alpha LXR isoform (Volle et al., 2004).

Two other recent reports showed that *Akr1b7* gene transcription in the small intestine, colon and liver was also controlled by bile acids through a FXR response elements (FXRE) located at position −292 to −280 on the promoter (Ge et al., 2011; Schmidt et al., 2011).

Several nuclear receptors, including FXR, pregnane X receptor (PXR) and constitutive androstane receptor (CAR), have been shown to protect against the cytotoxic effects of bile acids by increasing expression of binding proteins, transporters, and enzymes that detoxify bile acids (Zollner et al., 2006; Schmidt and Mangelsdorf, 2008). The combined use of KO mouse models for PXR/CAR receptors and pharmacological activators allowed identification of *Akr1b7* as one of their target genes in the liver (Liu et al., 2009; Table 4 and Figure 1).

Although molecular mechanisms mediating metabolic regulation of *Akr1b7* expression in the liver and intestine were profusely examined using genetic models or forced expression systems, there is no direct evidence for Akr1b7 expression in liver sections or in isolated hepatocytes, to date.

##### Detoxifying lipid aldehydes

Wild-type mice treated with PCN (pregnenolone-16α-carbonitrile; a PXR agonist) exhibited a decrease in malondialdehyde (MDA) levels (another by-product of polyunsaturated fatty acid peroxidation) compared with their vehicle-treated counterparts. The effect of PCN on MDA was abolished in *Pxr*^−/−^ mice. The authors suggested that PXR played a role in alleviating lipid peroxidation in small intestine. Since the induction of *Akr1b7* expression by PCN was also abolished by PXR KO, the effects of PXR on lipid peroxidation could be mediated, at least in part, by modulation of *Akr1b7* expression (Liu et al., 2009; Ge et al., 2011; Schmidt et al., 2011).

In light of the ability of Akr1b7 to reduce 4-HNE, Volle et al. have compared the status of lipid peroxidation in the small intestine of wild-type and *Lxr*α^−/−^ mice by measuring MDA levels. In the duodenum, a significant decrease in MDA concentrations was seen when wild-type mice were treated with T091317 (a synthetic agonist of LXR). This effect was not observed in *Lxr*α^−/−^ mice, where T091317 was unable to induce *Akr1b7*. The authors suggested that increased levels of LXRα in the small intestine and its activation by oxysterols could in turn, up-regulate the expression of detoxifying genes which could ultimately reduce oxidative stress (Volle et al., 2004). However, although the decrease in MDA contents is clearly a LXR dependent mechanism, the direct involvement of Akr1b7 seems unlikely. Indeed overexpression of Akr1b7 *in vivo* does not alter MDA levels (Ge et al., 2011).

##### Detoxifying bile acids or lipid peroxidation induced by bile acids excess (figure 5)

**Figure 5:**
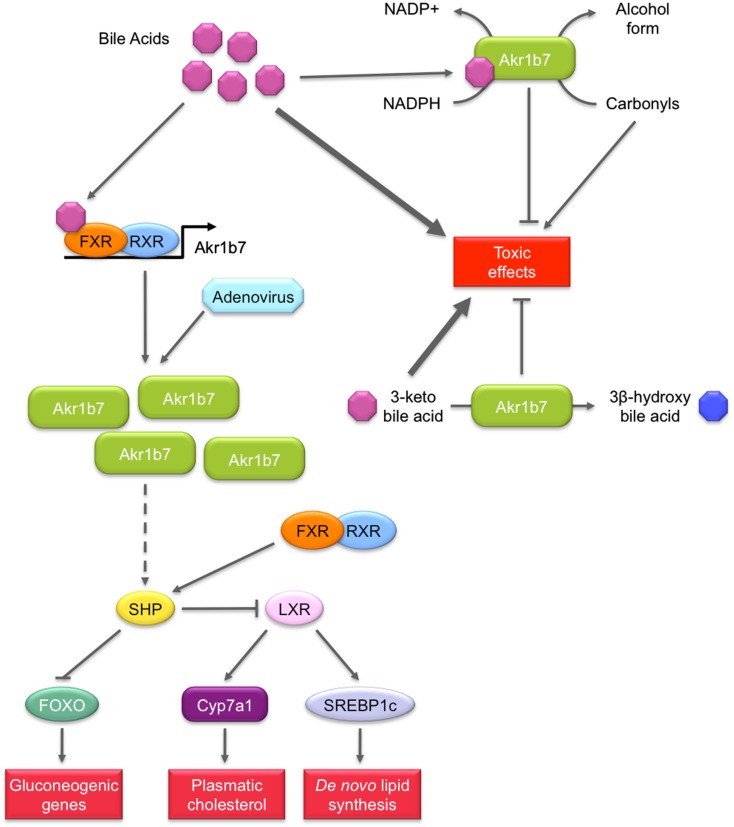
**Role of Akr1b7 in bile acids metabolism and signaling pathway**. Akr1b7 has the ability to reduce 3-keto bile acids to their less toxic 3β-hydroxy derivatives. In turn, bile acids can stimulate detoxification activity of Akr1b7, enhancing its capacity to reduce carbonyl and 3-keto bile acids. In addition to bile acids detoxification, Akr1b7 is also one of their target genes. In liver and intestine, bile acids induce the FXR-dependent *Akr1b7* transcription. The forced expression of Akr1b7 in mouse liver by the mean of recombinant adenovirus results in the down-regulation of gluconeogenesis and lipid metabolism. Mechanisms involved are not unravel yet but could rely on the up-regulation of SHP expression. Increased SHP levels could in turn repress both FOXO and LXR, leading to the inhibition of gluconeogenic genes and lipogenic genes, respectively. The mechanism by which Akr1b7 accumulation modulates the SHP gene expression appears independent from FXR and remains to be discovered (dashed lines).

Recently three independent studies proposed that *Akr1b7* or its rat ortholog *Akr1b14* could be involved in bile acid metabolism and/or signaling (Endo et al., 2011; Ge et al., 2011; Schmidt et al., 2011).

Bile acids are cholesterol-derived molecules produced for its solubilization in the gallbladder and intestine by forming mixed micelles with cholesterol and phospholipids. They are required for the activation of some pancreatic enzymes and for the absorption of cholesterol, lipid soluble vitamins and to a lesser extent, triglycerides and fatty acids from the intestine (Hylemon et al., 2009). They also act as signaling molecules by acting as ligands for several nuclear receptors including FXR and PXR or the membrane Gα_s_ protein-coupled receptors TGR5 as well as Gα_i_ protein-coupled receptors. Hence, bile acids contribute to the regulation of their own synthesis, fatty acid, lipid, and lipoprotein synthesis as well as glucose metabolism in the liver.

An *in vitro* enzymatic study using recombinant Akr1b14 (rat) and Akr1b7 (mouse) proteins showed that conjugated and unconjugated bile acids (chenodeoxycholic acid (CDCA)/glyco-CDCA and hyodeoxycolic acid (HDCA)/glyco-HDCA) specifically, quickly, and significantly activated the NADPH-linked reductase activity of Akr1b14 and to a lesser extent Akr1b7 activity (Endo et al., 2011). More accurately, higher specific activation of Akr1b14 was mediated by an interaction of bile acids with the His269 facilitating the release of NADP^+^. In Akr1b7, the histidine residue found at position 269 in the Akr1b14 protein is replaced by an arginine residue. This may account for weaker activation by bile acids. The authors suggested that bile acids could activate Akr1b14/Akr1b7 when their blood concentrations were elevated. As high concentrations of hydrophobic bile acids could induce cell injury through several pathways, e.g., lipid peroxidation, activation of Akr1b14 by bile acids may increase detoxification of the harmful lipid peroxidation by-products, and contribute to the attenuation of toxic effects of bile acids.

Because members of the AKR family (i.e., AKR1C4 and AKR1D1) were shown to be involved in the bile acid synthesis, Mangelsdorf’s group hypothesized that FXR-inducible Akr1b7 could be involved in bile acid metabolism in the intestine (Schmidt et al., 2011). They showed that Akr1b7 overexpression in heterologous HEK293 cells was associated with a reduction activity of 3-keto bile acids to their 3β-hydroxy derivatives, which are less toxic than their 3α-hydroxy epimers. Altogether, these results suggest a novel function for Akr1b7 in the detoxification of bile acids.

##### Possible role in metabolic action of bile acids signaling (figure 5)

In the liver, bile acids are able to influence glucose metabolism by at least two mechanisms. First, conjugated bile acids rapidly activate the insulin signaling pathway via Gα_i_ protein-coupled receptors or superoxide ions. In this aspect, they function much like insulin through the AKT pathway to activate glycogen synthase and repress neoglucogenic genes (Hylemon et al., 2009). Importantly, bile acids also mediate another control on liver metabolism through the transcriptional activation of the gene encoding the small heterodimer partner (SHP) via a functional FXRE in its promoter. The orphan receptor SHP can exert inhibitory interactions with forkhead box O1 (FOXO1), C/EBPα, and hepatocyte nuclear factor 4α transcription factors, known to activate neoglucogenic genes (Yamagata et al., 2004). The inhibitory control of bile acids on gluconeogenesis has also been demonstrated in FXR null mice or with the treatment of diabetic animals with the FXR agonist GW4064 (Ma et al., 2006; Zhang et al., 2006). SHP is also able to interact with LXR to down-regulate the gene encoding SREBP-1c which is the predominant transactivator for genes encoding enzymes involved in fatty acid, triglyceride, and VLDL biosynthesis (Horton et al., 2002).

These observations led Ge et al. (2011) to investigate the implication of FXR target gene *Akr1b7* in liver glucose and lipid metabolism using adenovirus-mediated Akr1b7 overexpression. Overexpression of hepatic Akr1b7 significantly reduced plasma glucose levels. This was associated with reduced hepatic mRNA levels of gluconeogenic genes encoding phosphoenolpyruvate carboxykinase and glucose 6-phosphatase and of peroxisome proliferator-activated receptor γ co-activator 1α, as well as markedly increased hepatic SHP mRNA levels. Hepatic lipogenesis was also affected: mRNA levels of SREBP-1c and several lipogenic genes including Fas, diacylglycerol acyltransferase 2, cholesterol 7α-hydroxylase, and the ATP binding cassette G5 were significantly reduced. Nevertheless other FXR target genes such as the bile salt export protein or the multi drug resistance 2 were not affected. This excluded the hypothesis that FXR was activated through overexpression of Akr1b7. Forced hepatic expression of Akr1b7 also significantly lowered plasma glucose and hepatic triglyceride and cholesterol levels in db/db mice. These results show that overexpression of Akr1b7 in the liver selectively down-regulates gluconeogenic and lipogenic gene expression, even though the molecular mechanisms linking Akr1b7 to these metabolic changes are not elucidated. Yet, we can speculate that up-regulation of the receptor SHP upon Akr1b7 overexpression has a central role in the coordinated repression of both gluconeogenic and lipogenic metabolisms.

Unlike overexpression of Akr1b8/AKR1B10 that leads to increased lipogenesis, overexpression of Akr1b7 in the liver reduced hepatic lipid accumulation suggesting that *in vivo* these closely related AKR1B proteins display non-overlapping functions. The possibility that other AR (including AKR1B10) endowed with 4-HNE reductase activity could modulate bile acid metabolism under the control of FXR, LXR, or PXR, remains to be determined. Transcripts for a novel murine ARLP (Akr1b16) were recently described in the liver but expression of Akr1b16 has not been investigated in the small intestine (Salabei et al., 2011). Moreover, mechanisms controlling their expression in these tissues have not yet been elucidated.

#### Akr1b3/AKR1B1: Altering (liver) metabolic capacity

Both Akr1b3 and AKR1B1 are ubiquitously expressed. *Akr1b3* transcripts were detected in the liver and small intestine at similar levels (Joshi et al., 2010). *AKR1B1* messenger RNAs are found in the liver and small intestine (Cao et al., 1998). Western blot analyses confirmed AKR1B1 expression in the small intestine although it was undetectable in the normal adult liver. However AKR1B1 expression could be induced by alcoholic cirrhosis (O’Connor et al., 1999).

**Figure 6:**
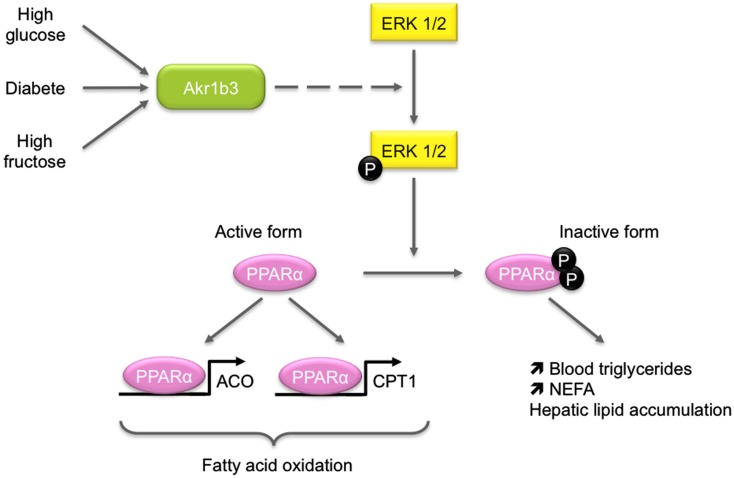
**Role of Akr1b3 in hepatic lipid homeostasis**. Diabetic conditions (high glucose or high fructose) lead to *Akr1b3* up-regulation and Akr1b3-dependent ERK1/2 activation and are often associated to dyslipidemia. The ERK1/2 mediated phosphorylation of PPARγ inhibits its transcriptional activity resulting in down-regulation of β-oxidation master genes. Impaired β-oxidation favors hepatic lipid accumulation and increases blood triglycerides and non-esterified fatty acids (NEFA). The mechanism by which Akr1b3 induces ERK1/2 phosphorylation is still unknown (dashed lines).

In diabetic mice, Akr1b3 is involved in glucose metabolism in some tissues through its participation in the polyol pathway. In response to chronic hyperglycemia, Akr1b3 catalyzes the rate-limiting reduction of glucose into sorbitol which is in turn converted into fructose by the sorbitol dehydrogenase (SDH). Activation of this pathway is involved in the development and progression of diabetic complications (Brownlee, 2001). Some evidences suggest that it could also be implicated in lipid metabolism. First, diabetic patients exhibit elevated blood triglycerides and non-esterified fatty acids and second, fructose-fed rats display an elevation of blood triglycerides and a reduction of peroxisome proliferator-activated receptor alpha (PPARα) activation (Roglans et al., 2007). These data led Qiu et al. to investigate the effects of hepatic activation of the Akr1b3/polyol pathway on lipid metabolism (Figure 6). In a murine hepatocyte cell line, overexpression of Akr1b3 was able to suppress PPARα transcriptional activity through extracellular signal-regulated kinase (ERK1/2)-mediated inactivating phosphorylations on Ser-12 and Ser-21. This resulted in decreased expression of genes involved in peroxisomal and mitochondrial β-oxidation pathway. In addition, Akr1b3 is up-regulated under high glucose concentrations in mouse hepatocyte AML12 cells and *in vivo* in diabetic mice. The genetic ablation or pharmacological inhibition of Akr1b3 in diabetic mice decreased ERK1/2-dependent phosphorylation of PPARα and blood triglycerides levels. In diabetic animals, SDH KO was accompanied by a reduction of blood triglycerides. This study clearly indicated that Akr1b3 and the polyol pathway sensed intracellular levels of glucose and adjusted PPARα activity through its phosphorylation/dephosphorylation, which in turn affected cellular lipid homeostasis (Qiu et al., 2008). In db/db mice, inhibition and knock-down of Akr1b3 induced the decrease of both plasma and liver triglycerides levels. Inhibition of Akr1b3 in db/db mice led to an improvement of hepatosteatosis due to the up-regulation of acetyl-coA oxidase and apolipoprotein A-V, two PPARα target genes involved in lipid catabolism (Qiu et al., 2012). Mechanisms by which Akr1b3 influences ERK-dependent inactivation of PPARα remain to be established.

AKR1B1 is also involved in the polyol pathway. Therefore, the relevance of these results in human remains to be determined. However, studies using *Akr1b3*^−/−^ mice or transgenic mice overexpressing AKR1B1 revealed no abnormalities in either the liver or small intestine (Yamaoka et al., 1995; Aida et al., 2000).

In addition to this possible implication in metabolism, AKR1B1 could be an actor of small intestine protection against electrophilic carbonyls. Its expression is also induced in human colon cancer cell lines SW-480 and HT-29, by proteasome inhibitors known to induce Nrf2 expression (Ebert et al., 2011). Characterization of *Akr1b3* promoter has shown that Nrf2 could regulate its activity through an antioxidant response element 1 (−953 to −945) and an AP1 site (Nishinaka and Yabe-Nishimura, 2005). The arrangement of these elements in the stress response region was conserved between *Akr1b3* and *AKR1B1* promoters (Figure 3).

Moreover, in a similar way as AKR1B10, AKR1B1 displayed enzymatic properties allowing protection of intestinal cells against dietary electrophilic carbonyls. Indeed, AKR1B1 kinetic parameters are compatible with reduction of glutathione-conjugated carbonyl compounds (Shen et al., 2011). Studies in healthy tissues could allow a better understanding of AKR1B1 implication in cytoprotection against oxidative damage.

### AKR1B in white adipose tissue

For decades, adipose tissue was considered an inert mass of energy storage in which adipocytes were unique in the quantity of lipid that they can store during period of energy excess and mobilize as free fatty acids when required. In fat pads, adipocytes are intermingled with other cells including blood cells, endothelial cells, adipose precursors of varying degree of differentiation, and fibroblasts (Ailhaud et al., 1992). Adipose tissue plays a crucial role in the regulation of energy homeostasis, insulin sensitivity, and lipid/carbohydrate metabolism. These actions are mediated by both non-secreted proteins and hormones produced by adipocytes. These adipokines have wide-ranging effect on energy intake, energy expenditure, carbohydrate and lipid metabolism, including nutrient partitioning, and fuel selection (Trayhurn and Beattie, 2001; Fonseca-Alaniz et al., 2007; Vázquez-Vela et al., 2008). White adipose tissue expansion takes place rapidly after birth and it retains some plasticity throughout life (Frühbeck, 2008). Adipose precursors are able to differentiate in mature adipocytes and acquire features of fully differentiated cells during adipogenesis. Acquisition of the adipocyte phenotype is characterized by chronological changes in the expression of numerous genes. Studies on 3T3-L1 cells, a line of murine preadipocytes that have the ability to accumulate lipids during their hormonal-induced differentiation into adipocytes, have allowed identification of C/EBPs and PPARγ as master regulators of adipogenesis (Tontonoz et al., 1994; Wang et al., 1995; Tanaka et al., 1997; Rosen et al., 1999). The earliest event of adipogenesis is the transient induction of C/EBPβ and C/EBPδ expression, which in turn induces PPARγ and C/EBPα expression (Tang et al., 2004). This is followed by the synergic action of PPARγ and C/EBPα that enhance the expression of several genes characterizing the adipocyte phenotype along with massive triglyceride accumulation (Wu et al., 1999). SCD1, phosphoenolpyruvate carboxykinase, Fas, ACCA, malic enzyme, insulin receptor, adipocyte specific fatty acid binding protein, leptin (Rosen and Spiegelman, 2000) are some of the essential genes induced during terminal differentiation of adipocytes. Adipogenesis is tightly regulated by a set of pro- and anti-adipogenic factors including insulin, FGF, EGF, and prostaglandins (Gregoire et al., 1998). Adipose tissue expansion is resulting from adipocyte hyperplasia (generated by an increased number of adipocytes) and/or hypertrophy (caused by an enlargement of adipocyte resulting from increased lipid accumulation). Disruption of the mechanisms that control adipose tissue homeostasis can result in massive expansion of the tissue as exemplified during development of obesity (Henry et al., 2012). One of the most exciting challenges in the field of adipose tissue homeostasis is to identify the key regulators of adipose tissue expansion, which would allow a better understanding of the mechanisms involved in the pathogenesis of adipose tissue disorders. Recent reports based on functional studies and identification of novel enzymatic properties suggest that some AKR1B enzymes can regulate adipose tissue homeostasis.

#### Akr1b7: a negative regulator of adipose expansion

Until 2003, there was no mention of AR expression in white adipose tissue. In a transcriptomic study, Moraes et al. reported for the first time the detection of *Akr1b7* mRNA in mouse white adipose tissue. In this study, the authors highlighted a decrease in *Akr1b7* expression in abdominal white fat tissue from diet-induced obese mice when compared with mice fed a standard diet (Moraes et al., 2003). This decrease reflected either the down-regulation of *Akr1b7* gene expression level per cell or the lower proportion of cells expressing the gene. Indeed, *Akr1b7* expression appeared to vary depending on the location of white adipose depots and was found enriched in the stromal vascular fraction (containing adipocyte progenitors) whereas it was virtually absent from mature adipocytes (Tirard et al., 2007). Accordingly, *Akr1b7* expression decreased during adipogenic differentiation of primary preadipocytes from the stromal vascular fraction and was detectable in the 3T3-L1 preadipocyte cell line.

The transcription factor SF-1, an essential regulator of *Akr1b7* expression in the adrenal cortex is absent from adipose tissue. Instead, adipocyte precursors express liver receptor homolog-1 (LRH-1; Clyne et al., 2002), a nuclear receptor that also binds to SF-1 regulatory elements and can substitute for SF-1 in tissues where it is not expressed (Sirianni et al., 2002). Consistent with decreased expression of Akr1b7 during adipogenesis, LRH-1 expression is rapidly down-regulated during adipocyte differentiation (Clyne et al., 2002). We thus evaluated the possibility that LRH-1 stimulated *Akr1b7* promoter activity in preadipocytes. Indeed, in co-transfection experiments in 3T3-L1 preadipocytes, LRH-1 induced *Akr1b7* promoter through the SF-1 binding site at −458 (unpublished observations). Therefore, the dynamic expression profile of LRH-1 may account for expression of *Akr1b7* in the preadipocytes-enriched stromal fraction (stromal vascular fraction) of various fat depots and its transitory expression in the early differentiation steps of 3T3-L1 cells.

Overexpression of Akr1b7 in 3T3-L1 preadipocytes prevented lipid droplets accumulation during adipogenic differentiation. In contrast, knock-down of Akr1b7 accelerated differentiation and lipid accumulation in 3T3-L1 cells. These results allowed us to demonstrate that Akr1b7 is a negative regulator of adipogenesis *in vitro*, which prevents differentiation by reducing lipid storage (Tirard et al., 2007). The mechanisms through which Akr1b7 inhibits adipocyte differentiation *in vivo* were recently unraveled by our group using *Akr1b7*^−/−^ mice. These will be discussed below.

**Figure 7:**
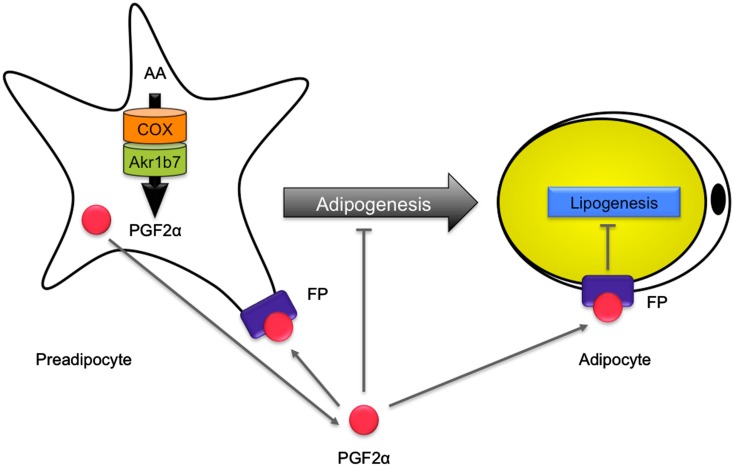
**Role of Akr1b7 in adipose tissue homeostasis**. In preadipocytes, arachidonic acid (AA) is metabolized into PGH_2_ by COX and is further converted into PGF_2α_ by the PGFS activity of Akr1b7. PGF_2α_ is supposed to mediate an autocrine activation of FP receptors on preadipocytes that inhibits their differentiation into adipocytes. In addition, PGF_2α_ inhibits lipogenesis in mature adipocytes. Accordingly, altered production of PGF_2α_ in *Akr1b7*^−/−^ mice leads to an expansion of adipose tissue due to both hyperplasia and hypertrophy of adipocytes.

Akr1b7 has two different enzymatic activities that could potentially be involved in the regulation of white adipose tissue homeostasis. First, just like Akr1b3, Akr1b7 is endowed with PGFS activity allowing synthesis of PGF_2α_, a potent inhibitor of adipogenesis (Serrero et al., 1992; Kabututu et al., 2009). Indeed, PGF_2α_ is able to inhibit differentiation of 3T3-L1 into adipocytes, through binding to the FP receptor. Activation of FP receptor in turn blocks PPARγ and C/EBPα expression through a Gα_q_-calcium-calcineurin-dependent signaling pathway (Liu and Clipstone, 2007). PGF_2α_ was also shown to block adipogenesis through activation of mitogen-activated protein kinase, resulting in inhibitory phosphorylation of PPARγ (Reginato et al., 1998). Second, Akr1b7 reduces 4-HNE, the accumulation of which is known to induce an excessive development of adipose tissue through adipocytes hypertrophy. Detailed analysis of *Akr1b7*^−/−^ mice allowed us to show that Akr1b7 behaved as an anti-adipogenic factor limiting white adipose tissue expansion, essentially through the regulation of PGF_2α_ levels *in vivo* (Volat et al., 2012; Figure 7).

*Akr1b7*^−/−^ mice displayed excessive basal adiposity resulting from both adipocyte hyperplasia and hypertrophy. They further exhibited increased sensitivity to diet-induced obesity. Following adipose enlargement and irrespective of the diet, they developed liver steatosis and progressive insulin-resistance. Akr1b7 loss was associated with decreased PGF_2α_ white adipose tissue contents. Cloprostenol (a PGF_2α_ agonist) administration in *Akr1b7*^−/−^ mice normalized white adipose tissue expansion by altering both *de novo* adipocyte differentiation and size. Treatment of 3T3-L1 adipocytes and *Akr1b7*^−/−^ mice with cloprostenol suggested that decreased adipocyte size resulted from inhibition of lipogenic gene expression. Hence, Akr1b7 is a major regulator of white adipose tissue development through at least two PGF_2α_-dependent mechanisms: inhibition of adipogenesis and lipogenesis (Volat et al., 2012).

#### Akr1b8/AKR1B10

Although we showed that Akr1b8 is devoid of PGH_2_ 9-,11-endoperoxide reductase activity (Kabututu et al., 2009), QTL mapping allowed identification of Akr1b8 as a possible regulator of adiposity in mouse (Derry et al., 2010). *Akr1b8*^−/−^ male mice had a tendency to be fatter than their wild-type littermates, under both standard and high fat diets. This was particularly obvious for the gonadal fat pad. Under high fat diet, *Akr1b8*^−/−^ mice also displayed higher serum cholesterol than their wild-type littermates. The mechanisms involving Akr1b8 in the regulation of white adipose tissue homeostasis remain completely unknown and are not necessarily resulting from expression of the gene in the adipose tissue. Indeed, we were unable to detect significant Akr1b8 protein expression in various adipose depots in mouse (Volat et al., 2012). Although we cannot exclude that Akr1b8 could be expressed in variable amounts depending on the mouse genetic background and the location of fat pads, it should be considered that the effect of Akr1b8 ablation could be indirect. Thus the involvement of Akr1b8 in adipose tissue homeostasis should be carefully examined with respect to its expression sites and enzymatic activities.

AKR1B10 expression in the adipose tissue has not yet been determined. Because AKR1B10 and Akr1b8 are not only detoxifying enzymes but also affect *de novo* fatty acids synthesis in cancer cells, it would be interesting to determine if and which of the enzymatic activities of AKR1B10 could be involved in adipose tissue homeostasis (Ma et al., 2008; Wang et al., 2009; Joshi et al., 2010).

#### Akr1b3/AKR1B1

Akr1b3 is expressed in undifferentiated 3T3-L1 preadipocytes and during the early phase of their differentiation. Akr1b3 can synthesize PGF_2α_ in 3T3-L1 cells, which inhibits their differentiation through stimulation of the FP receptor (Fujimori et al., 2010).

In addition to their PGF_2α_ synthase activity, Akr1b3 and AKR1B1 also catalyze the isomerization of PGH_2_ to PGD_2_ (Nagata et al., 2011). The impact of PGD_2_ on white adipose tissue homeostasis is still very disputed. Indeed, lipocalin-type prostaglandin D synthase (*L-PGDS*^−/−^) mice show hypertrophy of adipocytes (Ragolia et al., 2005). In contrast, the knock-down of L-PGDS decreases lipid accumulation in 3T3-L1 cells (Fujimori et al., 2007). In agreement with these observations, transgenic mice overexpressing (human hematopoietic-type prostaglandin D synthase (H-PGDS) overproduce PGD_2_ and show signs of obesity and pronounced adipogenesis on a high fat diet (Fujitani et al., 2010). These investigations suggest that PGD_2_ promotes adipogenesis *in vivo*.

Because of its involvement in the synthesis of PGF_2α_ and PGD_2_, the involvement of Akr1b3 in white adipose tissue physiology may result from a balance between both enzymatic activities. However the relevance of these data has not yet been studied *in vivo*. Indeed, no defect in adipose tissue homeostasis have been reported in *Akr1b3*^−/−^ mice and the prostaglandin contents of their fat depots have not been analyzed (Aida et al., 2000; Ho et al., 2000).

Although the murine AR Akr1b3 could potentially participate in the homeostatic maintenance of adipose tissue, there is no available information about its human ortholog AKR1B1. Previous studies using transgenic mice with constitutive overexpression of AKR1B1 did not report any effect on adipose tissue (Yamaoka et al., 1995). On the basis of its enzymatic properties (PGF_2α_ and PGD_2_ synthesis and reduction of 4-HNE), AKR1B1 could be considered as a potential actor of adipose tissue physiology (Kabututu et al., 2009; Nagata et al., 2011; Shen et al., 2011). However its expression pattern in human fat depots remains to be determined.

## Conclusive Remarks

Understanding physiological functions of AKR1B/Akr1b enzymes is a really challenging task. In that regard, review of the literature highlights the marked contrast between the abundance of enzymatic data and the small number of reports dedicated to functional studies in a physiological context. The main reason for this discrepancy is likely to be rooted in the functional redundancy resulting from the obvious structure conservation of the members of this enzyme family. Moreover, *Akr1b* genes being tandemly arranged on a same chromosome, classical genetic approaches to study multigene families that consist in combining KO models for each isoforms to obtain double or triple mutant mice are almost impossible to setup. Thus, alternative approaches to genetically disrupt *Akr1b* in various combinations and locations are mandatory to advance our understanding of their functions. Nuclease targeted invalidation or RNA interference approaches combined to existing KO models could present as valuable options.

Gain/loss-of-function models available for the Akr1b7 isoform revealed its implication in metabolic function and adipose tissue homeostasis. Beside the widely accepted idea that Akr1b are detoxification enzymes, several teams have now provided functional demonstration that some isoforms are endowed with the capacity to produce directly (prostaglandins) or indirectly (fatty acids) signal molecules. We think that further exploration of the functions of AKR1B/Akr1b enzymes must now take this dual potentiality into account.

## Conflict of Interest Statement

The authors declare that the research was conducted in the absence of any commercial or financial relationships that could be construed as a potential conflict of interest.
